# Reflections on having a ‘calling’ as a medical doctor

**DOI:** 10.1007/s40037-014-0112-5

**Published:** 2014-02-25

**Authors:** Michiel Westerman

**Affiliations:** School of Medical Sciences, VU University Medical Center, Amsterdam, The Netherlands

## The concept of calling

Throughout time, plentiful narratives have originated on individuals achieving astonishing things after receiving a ‘calling’ to a certain vocation. Examples range from the biblical calling of Moses in the desert to liberate the people of Israel from their oppression and slavery in Egypt [1] to Frodo Baggins being called to the quest aimed at destroying the ring of Sauron [2].

More recently and more scholarly, the concept of ‘calling’ or having a calling to a career in medicine is finding its way into the medical education domain. Perspectives on Medical Education recently published a paper on career choice among medical students and the influence of having a calling [3].

Varying definitions exist on ‘calling’ as a vocation, but that of Dik and Duffy [4] is well accepted, who define calling as: a career that involves having an external summons, provides a sense of meaning or purpose, and is used to help others in some capacity. Following this same line of research, Goodin et al. [5] report in this issue of Perspectives on Medical Education on a survey study into the association between having a calling as a medical student and commitment to a speciality and levels of self-efficacy. Goodin et al. illustrate that having a calling bolsters medical students who have lower levels of self-efficacy and that it is positively correlated to career commitment. Therefore, and based on other arguments, the authors appeal for the incorporation of the concept of calling into the dialogue on speciality choice and career commitment.

## Four reflections on the concept of calling

When contemplating upon the interesting concept of ‘calling’, I would like to present four reflections made on its implications and/or limitations. First of all, Duffy et al. identified a decrease of ‘calling’ among medical students between their first and third year of medical school. However, research on ‘calling’ and the influence thereof in further stages within the medical trajectory is absent [6]. For instance, the three major transitions within the medical education continuum, i.e. the transition to the clinical phase of training, the transition to residency, and the transition to new hospital consultant, are acknowledged as intense and stressful stages within the medical career [7]. Wilkie and Raffaeli [8] state that each transition involves a ‘fundamental re-examination of who and what we are, even if this processing is occurring at a largely unconscious level’. More specifically, in the transition from speciality training to hospital consultant, new consultants find themselves confronted with, and often overwhelmed by, a host of new challenges, such as final responsibility for patient care and novel non-clinical tasks relating to financial, leadership and management responsibilities [9, 10]. As a result, new consultants are forced to rapidly respond and adapt to these new circumstances and job requirements. Therefore, levels of job commitment and satisfaction, as well as self-efficacy, are often under attack during transitions. Thus, it might be interesting to investigate the concept of calling and the influence thereof during this and other transitions in the medical trajectory. Since it might be that individuals are forced to return to their original incentives, and thus possibly their ‘calling’, to achieve a career in medicine during this fundamental re-examination of themselves. And if a ‘calling’ can bolster individuals when their expertise or self-efficacy are under stress it could be worthwhile to steer on the construct of calling during mentoring or coaching in order to facilitate these stages.

My second reflection on the concept of ‘calling’ attributes its locus. Dik and Duffy [4] define calling as an external summons. However, this external summons has somehow become an internal feature of the individual. Goodin et al. also touch upon this paradox in their discussion by introducing the Organismic Integration Theory addressing the locus of individual motivation. This theory is in its turn related to the Self Determination Theory, a leading theory in research on motivation among medical students. Research on Self Determination Theory in medical education shows that intrinsic motivation leads to better learning and performance of medical students [11]. Motivation in its turn is a trending topic within the dialogue on selection of medical students or residents. Because it appears that calling and intrinsic motivation might be intertwined, it could be worthwhile to introduce the concept of ‘calling’ into this ongoing discussion.

A third reflection concerns the source of the calling. Whether or not a ‘calling’ has become an internal factor, it remains rooted in an external summons. However, to my knowledge it remains unknown whether this ‘calling’ derives from input of family members, childhood experiences, spiritual beliefs or other. Furthermore, it could be that perceiving a ‘calling’ differs from already identified influential factors on career choice within medicine [12]. Therefore, in my opinion it would be informative to gain more insight into how and why individuals come to experience a ‘calling’ to medicine in order to make better use from this potentially valuable construct.

My fourth and final reflection relates to the fact that one is being ‘called’ to the unknown without the certainty that you will reach the end. Medical students who perceive a ‘calling’ to a career in medicine frequently lack a sound depiction of what this will bring them and lack the guarantee that they will succeed. This is illustrated by the above-described transition to hospital consultant in which physicians only then experience what it is to function as a medical specialist and if this resonates with their calling. Being driven by a calling often entitles a journey into the unknown. Just like Frodo Baggins who said upon accepting his calling to bear the ring to Mordor: ‘I will take the Ring, though I do not know the way’ [2]. However, it is this same calling that can bolster individuals with lower levels of self-efficacy as illustrated by Goodin et al.

## The calling

I would like to end by calling upon educators, students, doctors and researchers to explore and further clarify the interesting concept of having a ‘calling’ in medicine.
